# Supplementary material for: Biomineralized Nano-Assemblies of Poly(Ethylene Glycol) Derivative with Lanthanide Ions as Ratiometric Fluorescence Sensors for Detection of Water and Fe3+ Ions
Source: Polymers (Basel). 2022 May 13;14(10):1997. doi: 10.3390/polym14101997 (PMC9146912; doi:10.3390/polym14101997)
Supplement: Supplementary file 1 [file polymers-14-01997-s001.zip › polymers-1706259-supplementary.pdf]

polymers

Biomineralized Nano-Assemblies of Poly(Ethylene Glycol) Derivative with Lanthanide Ions as Ratiometric Fluorescence Sensors for Detection of Water and Fe^{3+} Ions

Tong Chen [1,2] and Sanping Zhao [1,2,*]

[1] College of Materials Science and Engineering, Wuhan Textile University, Wuhan 430073, China
[2] State Key Laboratory of New Textile Materials and Advanced Processing Technologies, Wuhan Textile University, Wuhan 430073, China; chentong1997518@163.com (T.C.)
* Correspondence: zhaosanping@163.com; Tel./Fax: +86-27-87426559

Supplementary Information

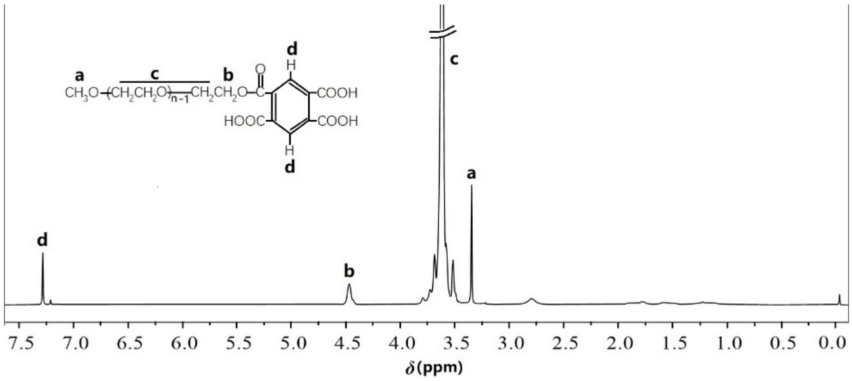

Figure S1. ^{1}H NMR spectrum of PMA-MPEG derivative in $CDCl_3$

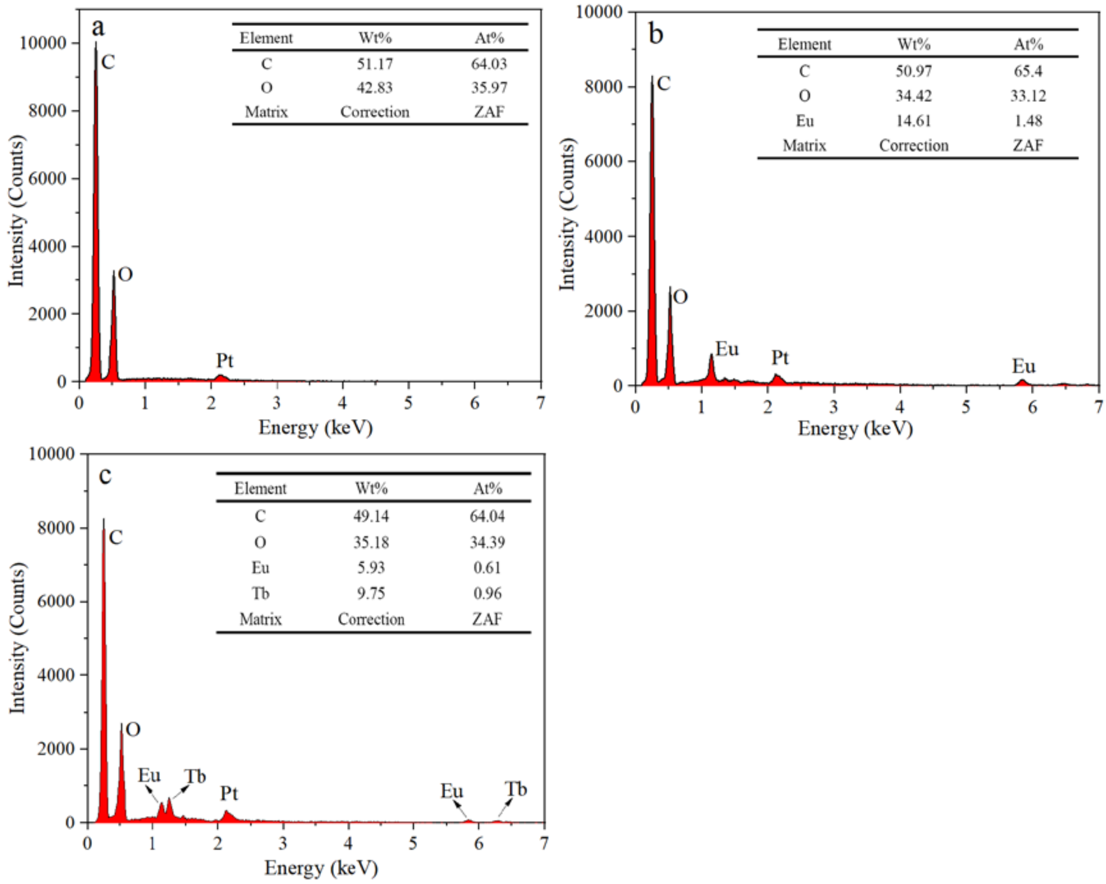

Figure S2 EDS analysis of $PMA-MPEG_{1.4}$ (a), Eu-PMA-MPEG (b) and Eu/Tb-PMA-MPEG (c).

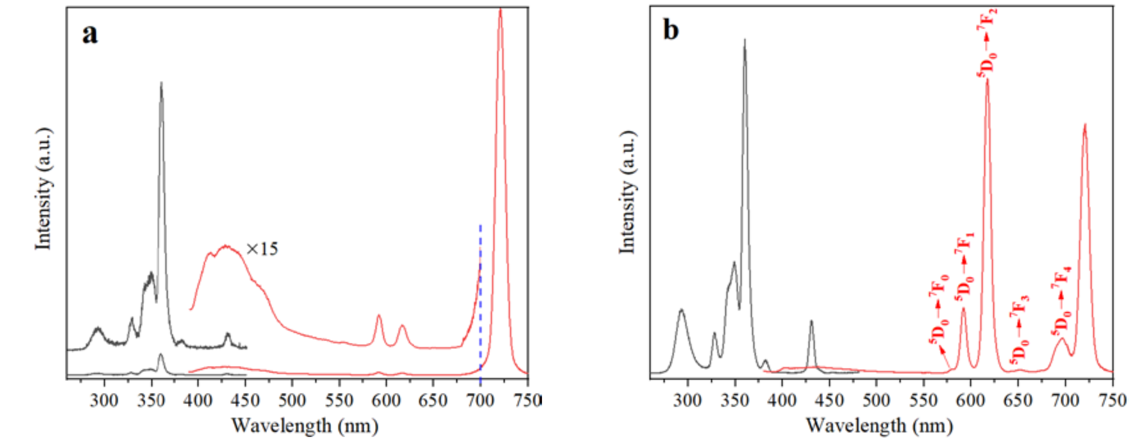

Figure S3 Excitation spectrum of Eu-PMA-MPEG monitored at 617 nm (black line), emission spectrum (red line) of Eu-PMA-MPEG in water (a) and in DMF (b), respectively.

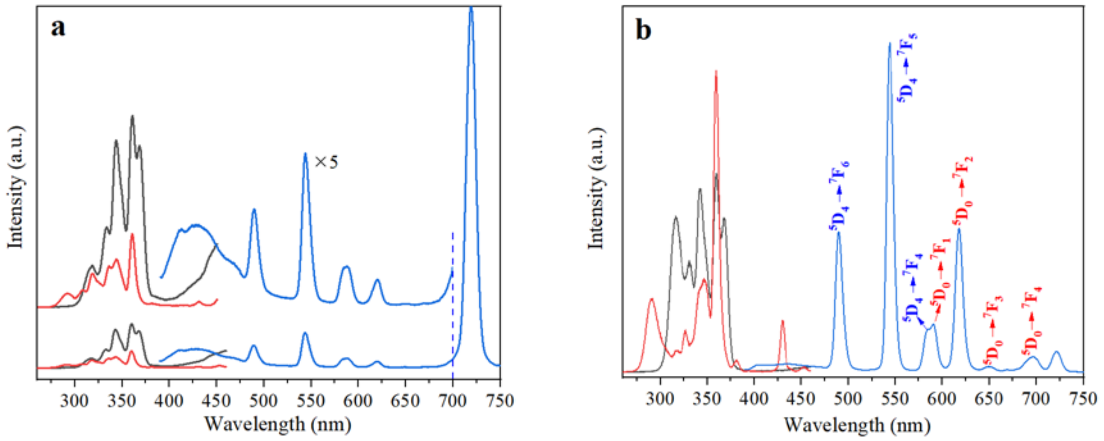

Figure S4 Excitation spectra of Eu/Tb-PMA-MPEG monitored at 544 nm (black line) and monitored at 617 nm (red line), emission spectrum (blue line) of Eu-PMA-MPEG in water (a) and in DMF (b), respectively.

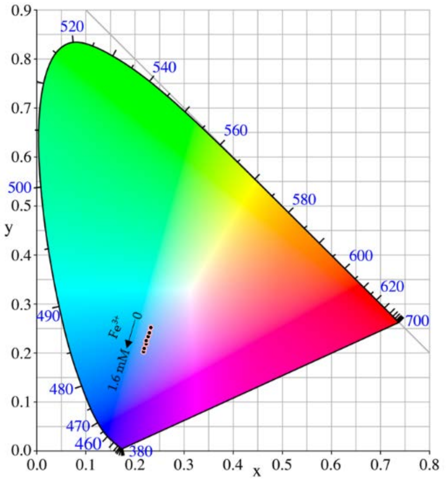

Figure S5 The CIE chromaticity diagram of Eu/Tb-PMA-MPEG solutions after treatment with various amounts of Fe^{3+} ions under the excitation wavelength of 360 nm
